# Renal protective effect of a hydration supplemented with magnesium in patients receiving cisplatin for head and neck cancer

**DOI:** 10.1186/s40463-018-0261-3

**Published:** 2018-02-02

**Authors:** Takahiro Kimura, Taijiro Ozawa, Nobuhiro Hanai, Hitoshi Hirakawa, Hidenori Suzuki, Hiroshi Hosoi, Yasuhisa Hasegawa

**Affiliations:** 1 Department of Otolaryngology-Head and Neck Surgery, Nara Medical University, Kashihara, Japan; 2 Department of Oto-Rhino-Laryngology, Toyohashi Municipal Hospi-tal, Toyohashi, Japan; 3 Department of Head and Neck Surgery, Aichi Cancer Center Hospital, 1-1 Kanokoden, Chikusaku, Nagoya, 464-8681 Japan; 4 Department of Otorhinolaryngology, Head and Neck Surgery, University of the Ryukyus, Nakazu, Japan

**Keywords:** Cisplatin, Nephrotoxicity, Magnesium, Head and neck cancer

## Abstract

**Background:**

Our study analyzes the effect of magnesium supplementation on nephrotoxicity in patients receiving cisplatin for head and neck cancer.

**Methods:**

We retrospectively reviewed the medical records of patients with head and neck cancer who received two doses of cisplatin (80 mg/m2) and 5-fluorouracil (800 mg/m2) 3 weeks apart from August 2008 to October 2012. The regimen prior to 2011 (crystalloid-only) involved the administration of 1000 mL of lactated Ringer’s solution on the day prior to cisplatin infusion and 2000 mL of continuous infusion of saline on the day of cisplatin infusion. The regimen after 2011 (magnesium-supplemented) did not involve hydration on the day before cisplatin administration but used 1000 mL of 0.9% saline with magnesium sulfate (20 mEq) administered for 3 hours before cisplatin infusion.

**Results:**

Sixty-five patients were treated with the crystalloid-only regimen and 56 patients with the magnesium-supplemented regimen. The mean creatinine clearance in the magnesium-supplemented group decreased by 4.9 mL/kg/min, whereas that in the crystalloid-only group decreased by 15.0 mL/kg/min after two courses. In multivariate analysis, only magnesium-supplemented hydration was an independent predictive factor for preventing cisplatin-induced nephrotoxicity (odds ratio = 0.157, 95% confidence interval 0.030–0.670, *P* = 0.0124).

**Conclusion:**

We demonstrated that an intravenous hydration regimen supplemented with magnesium prevented cisplatin-induced nephrotoxicity in patients with head and neck cancer.

## Background

Cisplatin was first administered to a cancer patient in 1971, and it became available for general oncology practice in 1978, first in testicular cancer and then in ovarian cancer [1]. Cisplatin also exerts a potent antineoplastic effect against head and neck cancer and is still a frequently used drug in this field. However, it has various side effects, such as gastrointestinal toxicity, myelosuppression, ototoxicity and nephrotoxicity. The nephrotoxic effect of cisplatin is, in particular, the most important dose-limiting factor in chemotherapy. Cisplatin-induced nephrotoxicity can be attributed to the impairment of proximal as well as distal tubular filtration and a severe progressive decrease in the glomerular filtration rate during treatment [2]. Hypomagnesemia was initially described in 1979 as an electrolyte abnormality induced by cisplatin chemotherapy [3]. Hypomagnesemia has been repeatedly confirmed by several studies. Additionally, Vokes, et al. demonstrated a high incidence of hypomagnesemia in cisplatin-treated head and neck cancer patients and its relation to the cumulative cisplatin dose [4]. A study using rats has shown a substantial additive effect of magnesium-depletion on renal toxicity induced by cisplatin. Therefore, attention must be paid to the aggravation of nephrotoxicity by magnesium-loss during cisplatin treatment especially in patients suffering from intense gastro-intestinal side effects [5]. Several studies have reported the prophylactic effect of magnesium supplementation on cisplatin-induced nephrotoxicity [6–8]. However, the study populations were relatively small and included patients with a history of previous treatments, surgery or chemotherapy.

To the best of our knowledge, there is no study has demonstrated the protective effect of magnesium supplementation against cisplatin-induced nephrotoxicity in patients with head and neck cancer. Here, we aimed to analyze the effect of magnesium supplementation on nephrotoxicity in patients receiving cisplatin for head and neck cancer.

## Methods

We retrospectively reviewed the medical records of patients with head and neck cancer who received cisplatin and 5-fluorouracil [5-FU] as an induction chemotherapy at the Department of Head and Neck Surgery of Aichi Cancer Center Hospital, Japan from August 2008 to October 2012. We selected patients who had no history of treatment for malignant tumors including head and neck cancer. The chemotherapy regimen consisted of 5-FU [800 mg/m^2^ continuous infusion from day 1 to 5] and cisplatin (80 mg/m^2^ on day 6) [9]. Chemotherapy was administered for two cycles 3 weeks apart.

**Fig. 1:**
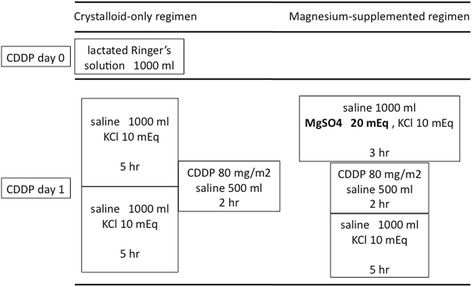
Change of hydration regimen in our study. In both regimens, dexamethasone (8 mg), granisetron (1 mg), and furosemide (20 mg) were given before cisplatin (CDDP) infusion. Post hydration was performed for 3 days with two liters of Soldem 3A® (Na 35 mEq/L, K 20 mEq/L, L-lactae 20 mEq/L, glucose 43.0 g/L)

The crystalloid-only regimen included the administration of 1000 mL of lactated Ringer’s solution on the day prior to the administration of cisplatin and 2000 mL of continuous infusion of saline on the day of cisplatin infusion. In April 2011, we changed the hydration regimen at our institution. The magnesium-supplemented regimen did not use lactated Ringer’s solution on the day before cisplatin, but instead it included 1000 mL of 0.9% saline with magnesium sulfate (20 mEq) for 3 hours before the administration of cisplatin (Fig. 1). The dose of magnesium sulfate was adopted from the cisplatin template based on the recommendations of the National Comprehensive Cancer Network. In both regimens, antiemetic agents (dexamethasone 8 mg, granisetron 1 mg) and a diuretic (furosemide 20 mg) were given, and post hydration was performed with two liters of Soldem 3A® (Na 35 mEq/L, K 20 mEq/L, L-lactate 20 mEq/L, and glucose 43.0 g/L) for 3 days after cisplatin infusion. However, while some patients in the crystalloid-only group were not given aprepitant, all patients in the magnesium-supplemented group were.

Renal damage was evaluated based on the reduction in creatinine clearance (Ccr) throughout the duration of chemotherapy. Ccr was calculated using the Cockcroft and Gault formula [10]. Nephrotoxicity was defined by a 20% reduction in Ccr after two courses of chemotherapy. Toxicities were graded using the Common Terminology Criteria for Adverse Events version 4.0.

Fisher’s exact test or Student’s *t*-test was used to compare the characteristics of patients before treatment. Student’s *t*-test was used to evaluate the differences in Ccr reduction between the crystalloid-only regimen and the magnesium-supplemented regimen. The contingency table with patient characteristics and nephrotoxicity was analyzed using Fisher’s exact test. Multivariate analyses were performed using a multiple regression model to investigate the relationship between magnesium supplementation and nephrotoxicity induced by cisplatin. The results were presented as odds ratio with 95% confidence intervals. *P* values < 0.05 were considered statistically significant. Analyses were performed with using JMP® 11 (SAS Institute Inc., Cary, NC, USA).

The study design was approved by the Institutional Ethics Committee and fulfilled the guidelines of the Declaration of Helsinki regarding the ethical principles for medical research.

## Results

The characteristics of all patients are shown in Table 1. There were 65 patients, 52 males and 13 females with a median age of 62 years, in the crystalloid-only group and 56 patients, 50 males and six females with a median age of 62 years, in the magnesium-supplemented group. There were no significant differences between the two groups regarding patient age, sex, performance status, body surface area (BSA), or hematocrit values. The magnesium-supplemented group had fewer cases with cancer of the nasal cavity and paranasal sinuses and oral cavity cancer and more cases of oropharyngeal cancer than the crystalloid-only group. There were no significant differences between the two groups in clinical parameters: serum levels of creatinine, albumin, and Ccr before chemotherapy. Because aprepitant was approved on October 16, 2009 in Japan, we only prescribed the antiemetic to 22 of 65 patients in the crystalloid-only group, but it was prescribed it to all patients in the magnesium-supplemented group.

**Table 1:** Patient characteristics

Characteristics		All (*n* = 121)	Crystalloid -only regimen (*n* = 65)	Magnesium –supplemented regimen (*n* = 56)	*P* value
Age	Median (range)	62 (28–78)	62 (28–77)	62 (42–78)	0.2943^†^
Sex	Male	102	52	50	0.1616^*^
	Female	19	13	6	
BSA (m2)	Median (range)	1.63 (1.27–2.00)	1.59 (1.33–1.96)	1.65 (1.27–2.00)	0.2542^†^
PS	0 / 1 / 2	67 / 46 / 8	38 / 22 / 5	29 / 24 / 3	0.5676*
Primary Site	Nasal cavity and Paranasal sinuses	15	14	1	0.0001^*^
	Oral cavity	4	4	0	
	Nasopharynx	1	0	1	
	Oropharynx	40	15	25	
	Hypopharynx	32	18	14	
	Laryynx	8	1	7	
	Cervical esophagus	13	7	6	
	Unknown	8	6	2	
Creatine (mg/dL)	Median (range)	0.71 (0.38–1.07)	0.69 (0.38–1.07)	0.73 (0.51–1.07)	0.1152^†^
Ccr (m L/min)	Median (range)	89.1 (54.5–186.7)	79.1 (54.5–156.1)	89.1 (57.0–143.6)	0.1712^†^
Albumin (g/mL)	Median (range)	4.1 (2.9–5.1)	4.1 (3.0–5.0)	4.2 (2.9–5.1)	0.1081^†^
Hematocrit (%)	Median (range)	39.7 (26.6–50.8)	39.1 (26.6–50.8)	40.4 (31.3–48.6)	0.1001^†^
Aprepitant	Administration	78	22	56	< 0.0001^†^

*BSA* body surface area, *Ccr* creatinine clearance

^*^Fisher’s exact test*;* ^†^Student’s *t*-test

**Fig. 2:**
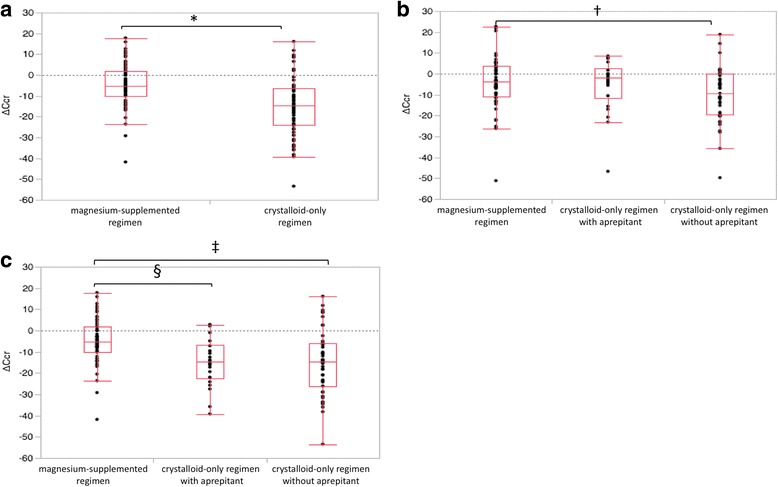
**a** Change in creatine clearance in the regimen groups after two courses (^*^p < 0.0001 Student’s t-test). **b** The crystalloid-only regimen group was divided into with and without aprepitant groups. Change in creatine clearance in the magnesium-supplemented regimen group and crystalloid-only group with and without aprepitant after one course (^†^*p* = 0.0298 Student’s t-test). **c** Change in creatine clearance in the three groups after two courses (^‡^p < 0.0001, ^§^*p* = 0.0026 Student’s t-test). ∆Ccr, difference in creatine clearance between chemotherapy courses

We noted significant differences in the change in Ccr (∆Ccr) between the magnesium-supplemented and crystalloid-only groups after treatment. The mean ∆Ccr after two courses of chemotherapy in the crystalloid-only regimen was 15.0 mL/min (standard error (SE), 1.7), and the mean ∆Ccr in the magnesium-supplemented group was 4.9 mL/min (SE, 1.4) (*p* < 0.0001). However, there was an obvious difference in the two groups regarding the administration of aprepitant, so we divided the crystalloid-only group into two groups with and without aprepitant. The incidence and severity of nausea and dehydration were worse in the group without aprepitant than in the group with aprepitant (Table 2). After one course of chemotherapy, there was no significant difference in ∆Ccr between the magnesium-supplement group (4.4 mL/min) and the crystalloid-only group with aprepitant (5.4 mL/min). Renal damage in patients in the crystalloid-only group without aprepitant was significantly more severe than in patients given the magnesium-supplemented regimen (10.1 mL/min, *p* < 0. 0298). However, after two courses of chemotherapy, patients in the magnesium-supplemented regimen group had lower occurrence of nephrotoxicity compared to patients in the crystalloid-only group with and without aprepitant. (4.9 vs. 14.7 and 15.1 mL/min, respectively) (Fig. 2).

**Table 2:** Toxicities occurring in patients treated with cisplatin and 5-FU

	Magnesium -supplemented regimen (n = 56)	Crystalloid-only regimen with aprepitant (*n* = 22)	Crystalloid-only regimen without aprepitant (*n* = 43)	*P* value*
Nausea				
Grade 1–2	30 (53.6%)	7 (31.8%)	29 (67.4%)	0.010
Grade 3	2 (3.6%)	1 (2.3%)	5 (11.6%)	
Vomitting				
Grade 1–2	2 (3.6%)	2 (4.6%)	6 (14.0%)	0.175
Grade 3	0 (0%)	0 (0%)	0 (0%)	
Dehydration				
Grade 1–2	5 (8.9%)	2 (4.7%)	12 (27.9%)	0.017
Grade 3	6 (10.7%)	2 (4.7%)	9 (20.9%)	

Toxicities were graded using the Common Terminology Criteria for Adverse Event version 4.0

*Chi-square test

We examined the relationship between nephrotoxicity induced by chemotherapy and clinical characteristics and parameters by univariate and multivariate analyses. Nephrotoxicity was defined as a 20% reduction in Ccr after chemotherapy. Nephrotoxicity was more strongly correlated with Ccr before chemotherapy, administration of aprepitant and regimen by univariate analysis (Table 3). Multivariate analyses were performed based on the results of univariate analysis. In multivariate analyses, only the magnesium-supplemented regimen of hydration was an independent predictive factor for protection against nephrotoxicity induced by cisplatin (odds ratio = 0.157, 95% confidence interval 0.030–0.670, *P* = 0.0124) (Table 4).

**Table 3:** Characteristics of patients classified by nephrotoxicity caused by cisplatin

Characteristics		Nephrotoxicity-	Nephrotoxicity+	*P* value^*^
Age	< 65	40 (42.6)	12 (44.4)	1.0000
	≥ 65	54 (57.4)	15 (55.6)	
Sex	female	13 (13.8)	6 (22.2)	0.3671
	male	81 (86.2)	21 (77.8)	
Primary site	Hypopharynx	23 (24.5)	9 (33.3)	0.4578
	Others	71 (75.5)	18 (66.7)	
Ccr	< 120 mL/min	86 (79.3)	22 (20.4)	0.1619
	≥ 120 mL/min	8 (61.2)	5 (38.4)	
Albumin	< 4 g/mL	32 (34.4)	12 (44.4)	0.3674
	≥ 4 g/mL	62 (66)	15 (55.6)	
Aprepitant	–	25 (26.6)	18 (66.7)	0.0002
	+	69 (73.4)	9 (33.3)	
Regimen	Crystalloid-only	41 (43.6)	24 (88.9)	< 0.0001
	Magnesium-supplemented	53 (56.4)	3 (11.1)	

Nephrotoxicity+ is defined as the 20% reduction in creatinine clearance after two courses

*Ccr* Creatinine clearance

^*^Fisher’s exact test

**Table 4:** Multivariate analysis of protective factors against nephrotoxicity induced by cisplatin

Protective factors	OR	95% CI	*P* value
Ccr (≥ 120/< 120 mL/min)	0.606	0.164–2.355	0.4567
Aprepitant (+/−)	0.525	0.161–1.563	0.2515
Regimen (magnesium-supplemented/crystalloid-only)	0.157	0.030–0.670	0.0124

*Ccr* Creatinine clearance, *OR* Odds ratio, *CI* Confidence interval. The multivariate analyses used a multiple regression model

## Discussion

The findings of this retrospective study showed that a magnesium-supplemented regimen of hydration reduces nephrotoxicity induced by cisplatin in patients with head and neck cancer. In univariate analysis, the characteristics of patients such as age, sex, primary tumor site and laboratory data were not associated with renal damage but change in the hydration regimen and the use of aprepitant were protective against renal dysfunction. To prevent intense gastro intestinal side effects, it is currently recommended that patients who receive cisplatin be given a three-drug combination of a 5-HT3 receptor antagonist, dexamethasone, and a neurokinin 1 receptor antagonist, such as aprepitant [11]. Patients with head and neck cancer are susceptible to dehydration, and chemotherapy-induced nausea and vomiting can aggravate dehydration. In Japan, aprepitant has been available since October 16, 2009. Administration of aprepitant prevented or reduced nausea and consequently mitigated dehydration. The patients in the magnesium-supplemented group benefited from the antiemetics more than those in the crystalloid-only group. However, in multivariate analysis, the magnesium-supplemented regimen was an independent predictive factor for protection against nephrotoxicity induced by cisplatin.

We hypothesize that supplementation with magnesium before cisplatin infusion protects against renal damage. Renal damage due to cisplatin involves a pathological change in the kidney that is characterized by focal acute tubular necrosis, primarily in the distal convoluted tubule [12]. This morphological change decreases tubular reabsorption, resulting in hypomagnesemia. It is unclear whether the depletion of serum magnesium aggravates cisplatin-induced nephrotoxicity, but previous studies have indicated some possibility of this relationship. Organic cation transporter 2, which regulates the uptake of cisplatin, is up-regulated under hypomagnesemia, and the renal accumulation of cisplatin is markedly increased [13, 14]. Several studies have reported the nephroprotective effect of magnesium supplementation during chemotherapy with cisplatin [6–8]. Willox J. C, et al. reported that all patients receiving cisplatin for testicular cancer have depleted serum levels of magnesium, indicating the protective effect of magnesium supplementation against cisplatin-induced renal tubular damage by measuring urine N-acetyl-β-D-glucosaminidase (NAG) [6] . We did not measure serum magnesium level or urine NAG, which constitutes a limitation of this study. There was no difference in the reduction in Ccr between the magnesium-supplemented group and the crystalloid-only group with aprepitant after one course. After two courses of chemotherapy, however, Ccr significantly decreased in the crystalloid-only group with aprepitant compared to the magnesium-supplemented group. This may be because a dysfunction in magnesium reabsorption after one course promotes cisplatin-induced nephrotoxicity in the second course so that the magnesium-supplemented regimen results in significantly better outcomes after the second course.

Several studies investigating the method of supplementation of magnesium during cisplatin treatment have recommended intravenous or oral supplementation or both [15]. Approximately 50% of the infused magnesium is excreted in the urine. Plasma magnesium concentration inhibits magnesium reabsorption in the loop of Henle [16]. When magnesium is intravenously administered, an abrupt but temporary elevation in the plasma magnesium concentration partially inhibits the stimulus for magnesium reabsorption in the loop of Henle. Magnesium uptake by the cells is slow, and therefore adequate replenishment requires sustained correction of hypomagnesemia. Martin et al. assigned 41 patients into groups with no magnesium supplementation, intravenous magnesium supplementation and oral supplementation during four courses of 100 mg/m2 cisplatin treatment. The study showed that both intravenous and oral magnesium supplementation can be efficacious in the prevention of cisplatin-induced hypomagnesemia. However, two patients treated with oral magnesium developed mild gastrointestinal symptoms (emesis and diarrhea), probably from the magnesium therapy. In contrast, none of the patients with intravenous magnesium supplementation showed these symptoms [17]. Because the location of tumors can make it difficult for patients with head and neck cancer to take oral drugs, we propose that magnesium should be administered intravenously before cisplatin infusion.

In the crystalloid-only regimen, saline was administered at a slower pace and more persistently during cisplatin treatment than that in the magnesium-supplemented regimen in which one liter of saline was rapidly administered within 3 hours before the infusion of cisplatin. Saline has a high concentration of chloride ions that prevents the substitution of water for chloride ions in cisplatin, thereby reducing the formation of aquated species of cisplatin that induce necrosis in tubule cells [18]. High volume hydration with saline before and after cisplatin injection is used to lower the concentration and to shorten the period of direct cisplatin exposure [19, 20].

There are several limitations in this study, such as its retrospective design and the limited availability of some clinical data. This study could not provide evidence for the linear relationship between the grade of hypomagnesemia and increase in the risk of nephrotoxicity induced by cisplatin. However, the population in this study is larger than that in the previous studies, and it is valuable to know that none of the patients had a history of anticancer treatment before chemotherapy.

## Conclusions

In conclusion, we demonstrated that an intravenous hydration regimen supplemented with magnesium has inhibitory effect on nephrotoxicity induced by cisplatin in patients with head and neck cancer.

## Abbreviations

∆Ccr: Decrease in Ccr
5-FU: 5-fluorouracil
BSA: Body surface area
Ccr: Creatinine clearance
CDDP: Cisplatin
CI: Confidence interval
OR: Odds ratio
SE: Standard error
